# Health professionals’ and leaders’ views on routine using patient-centered outcome measures in a Chinese palliative care unit: A qualitative study

**DOI:** 10.1017/S1478951525100369

**Published:** 2025-08-26

**Authors:** Yunyun Dai, Barbara A. Daveson, Jinfeng Ding, Yongyi Chen, Junchen Guo, Yu Cheng, Claire Johnson

**Affiliations:** 1 Faculty of Science, Medicine and Health, University of Wollongong, Wollongong, NSW, Australia; 2 School of Nursing, Guilin Medical University, Guilin, GX, China; 3 Palliative Care Outcomes Collaboration; Faculty of Science, Medicine and Health; University of Wollongong, Sydney, NSW, Australia; 4 Xiangya School of Nursing, Central South University, Changsha, HN, China; 5 Hunan Cancer Hospital, The Affiliated Cancer Hospital of Xiangya School of Medicine, Central South University, Changsha, HN, China; 6 The Nursing Department, Jinxin Geriatric Hospital, Chengdu, SC, China; 7 Palliative Aged Care Outcomes Program, Faculty of Science, Medicine and Health, University of Wollongong, Wollongong, NSW, Australia; 8 Monash Nursing and Midwifery, Monash University, Melbourne, VIC, Australia

**Keywords:** Outcome measurement, Barriers, Facilitators, Hospital-based palliative care, Implementation science

## Abstract

**Background:**

A person-centered outcomes-based quality improvement program is lacking within palliative care in Mainland China. The well-established Australian Palliative Care Outcome Collaboration (PCOC) national model improves palliative care quality.

**Objectives:**

This study aimed to explore the barriers and facilitators perceived by healthcare providers to integrating the PCOC model in a Chinese hospital-based palliative care unit.

**Methods:**

A qualitative descriptive study was conducted using semi-structured focus group and individual interviews. A rapid deductive analysis approach was selected for data analysis. The Consolidated Framework for Implementation Research framework was used to guide the study design, data collection, analysis, and interpretation.

**Results:**

Eighteen healthcare professionals participated in this study, four focus group interviews and five individual interviews were completed. Barriers to the PCOC integration included clinical application and workload concerns (patients in terminal stage, patients’ dialects, workload concerns, and staff shortages); attitudinal barriers (negative attitudes toward PCOC); psychological barriers (numbness to their work) and barriers related to knowledge and self-efficacy (lack of knowledge, capacity, and self-efficacy in palliative care). Facilitators included adapting the program to local contexts, ongoing education and feedback, effective PCOC data use, a supportive work and clinical environment and staff’s perceived advantages of the model across clinical, research and process domains.

**Significance of Results:**

The successful integration of the PCOC program hinges on local adaptation, improved data utilization, education, and IT support. In regions with less developed palliative care, enhancing professionals’ knowledge and self-efficacy is crucial. Incorporating assessment and clinical response protocols into technology can accelerate palliative care development and implementation.

## Introduction

Patient-centered Outcome Measures (PCOMs), including patient-reported and proxy-reported outcomes measures, are standardized and validated questionnaires that identify a patient’s primary health-related concerns(Etkind et al. 2015). The integration of PCOMs into routine clinical practice is a fundamental aspect of person-centered care within palliative care clinical practice. Evidence suggests that routine use of PCOMs enhances palliative care quality both at individual and organizational levels through better communication, identification and monitoring of needs, and enhanced care planning, decision-making and delivery(Currow et al. 2015). The effectiveness of PCOMs in palliative care is well-documented, leading to their integration into quality improvement and benchmarking initiatives in many Western countries (Bausewein et al 2016; Currow et al. 2015).

With a growing elderly population and an increasing incidence of cancer and chronic diseases(Tan et al. 2018; Wang et al. 2023), more and more Chinese people are seeking a “good”death and support at the end of their life, the demand for palliative and end-of-life care is on the rise in China(He et al. 2022; Tan et al. 2018). In response, the Chinese government began promoting palliative care in 2017, expanding to 185 cities in 2023 through a series of pilot programs. Despite these efforts, the delivery of palliative care lacks a national standardized quality evaluation and improvement framework, highlighting the need for an effective quality improvement model suitable for the Chinese context.

The Palliative Care Outcomes Collaboration (PCOC) is a national initiative based on PCOMs that has demonstrated statistically and clinically significant improvements in patient outcomes (Daveson et al. 2023). Funded by the Australian Government Department of Health and Aged Care, PCOC is designed to evaluate and support continuous improvement of palliative care outcomes through a quality feedback loop based on ongoing point-of-care assessment of individual patients’ and carers’ palliative care needs. PCOC supports continuous improvement in patient outcomes through a quality feedback loop, which includes routine assessment of patients’ and carers’ needs using standardized tools (Supplementary Figure 1) (PCOC 2023). Additionally, PCOC provides bi-annual quality reports to participating services, comparing clinical outcomes with national benchmarks and highlighting improvement areas (Supplementary Figure 2).

With the international success of the PCOC model, there’s interest in implementing it within a palliative care unit at a Chinese cancer hospital. We chose a cancer hospital for the pilot study because cancer patients constitute the majority of palliative care patients, and many palliative care units in Mainland China are either established within or embedded in cancer departments (Lu et al. 2018; Zhang et al. 2022). However, integrating such complex interventions into routine practice is challenging. Evidence suggests less than 20% of evidence-based interventions achieve successful integration in healthcare settings (Kilbourne et al. 2019). The gap between evidence and widespread usage often occurs as a result of overlooking contextual barriers before implementation (Nilsen 2015). Implementation science, focusing on promoting the adoption of innovations in routine practice, offers various frameworks and tools, such as the Consolidated Framework for Implementation Research (CFIR) to identify barriers to implementation (Damschroder et al. 2009).

Research on integrating PCOMs into routine practice, primarily from Western settings, points to varying barriers and facilitators across healthcare systems. There’s a lack of research in Mainland China, where quality improvement based on PCOMs is relatively new. To adapt the PCOC model effectively in Chinese clinical settings, it’s essential to understand healthcare providers’ perspectives on its implementation. This study aimed to investigate the barriers and facilitators to integrating the PCOC model perceived by healthcare providers in a palliative care unit based on the CFIR.

## Methods

### Study design

This qualitative study utilized semi-structured focus group and in-depth individual interviews to investigate healthcare providers’ views on the barriers and facilitators of implementing the PCOC model, accommodating participant time constraints. Separate focus groups for nurses and doctors collected their unique perspectives, and individual interviews were conducted with leaders and managers.

### Study settings

The study took place in a palliative care unit at a Chinese Cancer Hospital, which services 1590 inpatient beds and provides extensive outpatients services for a population of around 450,000 annually. The hospital offers treatments for all types of cancers from initial diagnosis through to palliative care and death. The palliative care unit has 22 inpatients beds and is staffed by12 nurses and 4 doctors. It has been a national leader in hospital-based palliative care since 2013, preceding the government’s palliative care initiative by four years. Although patients receive palliative care in the ward, most people die at home and are cared for by their families. Before the study, the hospital aimed to integrate the PCOC program into its clinical practice. To support this integration, PCOC education was provided through two 45-minute on-site sessions and a series of field case studies at the palliative care unit. The educational content included an introduction to the PCOC, detailed instructions on using the PCOC assessment tools and an overview of the clinical response framework.

### Participants

All 16 clinicians from the palliative care unit, along with two hospital leaders who are involved in the unit’s development and knowledgeable about the barriers and facilitators of PCOC implementation, were invited to the study. Written informed consent was obtained from all participants before interviews commenced. Participants were advised that taking part was voluntary, and they had the right to withdraw at any point before the interview transcripts were de-identified. A decision to not participate or withdraw had no impact on their relationship with the researcher, their colleagues, the palliative care unit or the hospital. All the data were de-identified after transcription and replaced with a study ID to ensure confidentiality.

### Interview guideline

The interview guide was developed based on the Consolidated Framework for Implementation Research (CFIR) (Damschroder et al. 2009), and was finalized by the research team (Supplementary Table 1).

### Data collection

Interviews were conducted two months post-PCOC education. Authors YD and JG trained by experienced qualitative researchers, pilot tested the interview guideline with three nurses from another department. All interviews were conducted either face-to-face in the palliative care unit’s meeting room or via online video meetings and held in a private setting with only the interviewers and interviewees present, ensuring a quiet and uninterrupted environment. The interviews were audio recorded without additional field notes.

### Data analysis

A rapid CFIR-based deductive analysis approach (directed content analysis) was selected for its time and resource efficiency (Johnson and Vindrola-Padros 2017; Taylor et al. 2018), providing timely yet effective and rigorous findings (Gale et al. 2019; Keith et al. 2017). The team developed a summary template with CFIR domains and neutral names for each interview question (Supplementary Table 2), enabling direct coding from recordings without transcripts, and supporting quotes were provided alongside. This method has proven effective, rigorous, and cost-saving (Nevedal et al. 2021). The research team, consisting of YD (PhD candidate in palliative care with five years’ experience in qualitative research), JG (PhD candidate in palliative care with three years’ experience in conducting interviews and data analysis), and YCh2 (master’s degree in nursing with specific training in qualitative data analysis methods), pilot tested this template on an interview audio recording. To ensure quality, pairs of researchers independently analyzed each interview (YD and JG, YD and YCh2), resolving discrepancies through discussion. Finally, all summary points were systematically organized into a matrix according to the roles of the participant – doctors, nurses, and managers – and were translated into English at this stage (Supplementary Table 3).

## Results

### Characteristics of participants

In this study, all 16 clinicians from the palliative care unit and two hospital leaders participated, engaging in either four focus group interviews (with 11 participants total, 2–4 participants per group) or five individual interviews. Of the 16 clinicians, 12 were nurses and four were doctors. The majority of participants were female (n = 17, 94.4%), with an average age of 42.3 years (ranging from 27 to 63 years) and 1 to 10 years of palliative care experience.

### Qualitative interview findings

**Figure 1.**
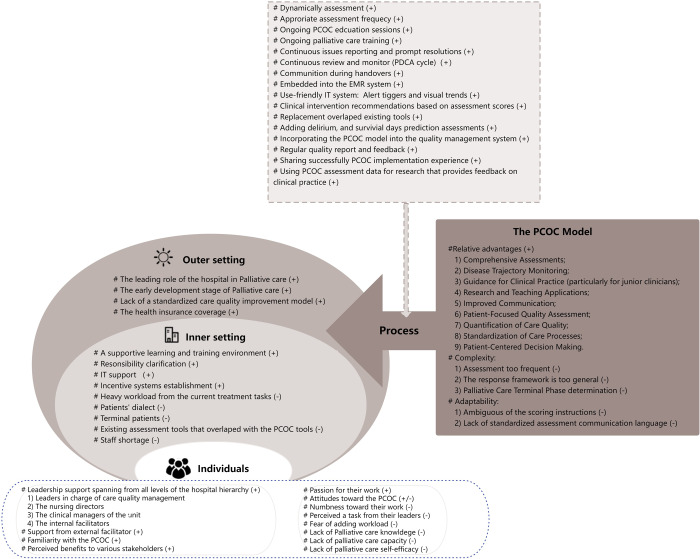
Barriers and facilitators to the PCOC integration. Note: +: Facilitators, -: Barries; PCOC: Palliative Care Outcome Collaboration.

Individual interviews averaged 44 minutes, and focus groups took about 68 minutes. Figure 1 outlines barriers, facilitators, and strategies for PCOC integration, based on CFIR constructs: 17 barriers, 15 facilitators, and 16 recommended strategies were identified. These constructs provided evidence and guidance for future PCOC implementation.

#### Innovation domain

##### Innovation evidence-base and its relative advantage

Following the education and use of the PCOC model for two months, participants acknowledged the potential benefits of this model. They highlighted several key advantages of the PCOC program: 1) Comprehensive Assessments; 2) Disease Trajectory Monitoring; 3) Guidance for Clinical Practice (particularly for junior clinicians); 4) Research and Teaching Applications; 5) Improved Communication; 6) Patient-Focused Quality Assessment; 7) Quantification of Care Quality; 8) Standardization of Care Processes; 9) Patient-Centered Decision Making. *For instance, a manager expressed the unique value of the PCOC program by stating, “Current quality management focuses on processes, policies, and patient satisfaction surveys, while the PCOC program concentrates on patients’ clinical outcomes, aligns more closely with the concerns, and needs of the patients themselves, highlighting the distinctive features of quality management in specialized palliative care.”*

##### Innovation complexity

Clinicians find the PCOC tools user-friendly but are concerned about the frequency of assessments, feeling once a day is too much and increases their workload. “*Once a day is too much for us, it could add our workload.*” (Nurses’ focus group) This was a prominent theme expressed in both focus group interviews with doctors and nurses and in two in-depth individual interviews where the sentiment was echoed: “*It is impossible to assess a patient once a day.*” Furthermore, nurses were concerned there are: “*Too many items, for example, if a patient suffers from nausea in the morning and I assess it, then pain occurs in the afternoon, I need to assess all the items again*.” (Nurses’ focus groups)

##### Innovation adaptability

Ensuring the PCOC program suits the current context is essential for its successful integration into routine clinical practice. Participants emphasized two key areas that require attention: modification to the PCOC program itself, and its implementation process.

For the program, participants pointed out the need for more specific scoring instructions, and a detailed description of appropriate interventions corresponding to assessment scores, one nurse highlighted the issue and stating *“The PCOC program’s response framework is too broad and misses tailored interventions. Like, for a SAS-pain score of 7 out of 10, we need clear intervention recommendations.”*

Additionally, doctors suggested the inclusion of a survival predictive assessment scale to enhance the accuracy of determining the Palliative Care Phase. As one doctor noted, *“If a survival predictive assessment scale is there for the Palliative Care Phase, it would be more accurate for us to determine the Terminal Phase.”*

Clinicians also proposed several changes to the implementation process to improve the utility of the PCOC measures in their clinical practice. For instance, they suggested modifying the assessment of frequencies to align with the unit’s typical practices; implementing standardized communication methods to guide clinicians; presenting the PCOC assessment scores as visual trends; and reporting the patient’s palliative care phases during handovers.

#### Individuals’ domain

##### Roles subdomain _ leaders and implementation facilitators

Participants emphasized the role of leadership in the program’s implementation, suggesting actions like leaders’ review, monitoring, and random inspections to ensure the accuracy of PCOC assessment. One nurse underscored this point by stating, *“It is important for the leaders to review the PCOC program implementation process and provide feedback during the handovers.”*

Clinician also stressed the importance of PCOC education on the benefits of the program, and recommended incorporating the PCOC quality report into the hospital’s quality management system, this approach not only highlight the program’s significance but also guarantee its sustainability.

There were different opinions regarding the quality manager for the PCOC program. Two focus groups and an individual advocated for appointing a designated quality manager who possess deep knowledge of the PCOC program, and expertise in identifying issues, providing feedback, and guiding effective patient assessment. However, two other focus groups preferred a collective quality management approach, involving all staff rather than relying on a single internal facilitator.

Regarding external facilitators, participants highlighted the need for flexible PCOC education and integrating the PCOC assessment tools into the hospital’s IT system in a user-friendly manner. They recommended continuous support throughout the implementation process, including regular feedback and effective use of assessment data. Sharing experiences from successful Australian implementations was also valued.

##### Characteristics subdomain _ capability and motivation

Some participants indicated that their familiarity with the PCOC program wasn’t a barrier to its implementation. However, they felt that their views on and capacity in palliative care posed significant challenges. One nurse mentioned, “*Using the PCOC tools won’t be hard; we’ll get familiar with it through some training sessions.*” In contrast, other participants, particularly senior clinicians, highlighted that a lack of knowledge of the PCOC program could make them reluctant to use it. These individuals found it challenging to embrace new practices and were concerned about the potential increase in workload.

Participants stressed the importance of palliative care training to improve their knowledge, self-efficacy, and capacity in palliative care to facilitate the PCOC program. As one nurse in the focus group stated, *“We’ll think about using something like the PCOC program to improve our care only if we’re equipped for palliative care. It’s crucial we get palliative care training and refresh our views on it.”* This sentiment was echoed by doctors, and further reinforced in an individual interview with a nurse.

Despite these obstacles, participants were motivated to adopt the PCOC program due to its benefits for stakeholders, its effectiveness, and their professional dedication. However, factors like work numbness, negative views on PCOC, and seeing it as a leader-imposed task could hinder its usage. Some believed that gaining a better understanding of the PCOC could improve their attitudes towards it. As one nurse noted, *“The PCOC program can provide me with a patient’s disease trajectory, enabling me to deliver better care, this, in turn, benefits the patients and motivate me to use the program.”* A similar sentiment was expressed in the doctors’ focus group.

#### Internal settings domain

##### Culture and compatibility

In terms of internal setting factors, participants emphasized the importance of creating a supportive learning environment and implementing the PCOC program hospital-wide, aiming to make its integration a routine responsibility for all staff and promoting a shared commitment to its use. One nurse highlighted this by stating, *“I think it is necessary to roll out the PCOC program across our hospital, making us feel that it is our routine responsibility, meanwhile, we can also communicate how to use this program effectively.”*

However, participants also expressed concern about the number of assessment tools used within their unit, compounded by staff shortage, could significantly increase workload. These concerns were pronounced given the nature of treatment work in the palliative care unit in Mainland China, which clinicians felt differed from practices in other countries. A doctor expressed this sentiment by stating, *“We clinicians are already fully occupied with treatment tasks in our unit, it would add too much workload to our daily practice. This situation might differ from palliative care units in other countries, where the volume of treatment work may not be as high.”*

Another concern raised by clinicians was the potential resistance from terminal patients, as well as the barriers posed by patients who speak different dialects. *A nurse commented on the former issue, noting, “Some patients might feel annoyed if we assess them too often, especially those who are terminally ill or whose symptoms are under control.”*

To ensure the PCOC program integrates smoothly into the current workflow, clinicians suggested several strategies. One key recommendation was to replace any existing assessment tools that duplicate the functions of the PCOC tools, thereby streamlining the assessment process. Clinicians also emphasized the need to clearly clarify the responsibilities, this was highlighted in a focus group where a doctor questioned, *“Who do the assessment, doctors or nurses?”* Meanwhile, they stressed the importance of determining the appropriate frequency of the PCOC assessments. A nurse explained, *“We already assess patients dynamically, so using PCOC tools dynamically matters too. But, we need to figure out the right frequency to not overload our work.” This concern was e*mphasized many times by other nurses and echoed by managers.

In addition to these structural adjustments, participants across different interviews mentioned the importance of communicating assessment results during handovers, conducting ongoing PCOC program education sessions, and applying the Plan-Do-Check-Act (PDCA) cycle to dynamically review the implementation of the PCOC program as key strategies to facilitate its integration.

##### Information technology infrastructure

All the participants agreed that IT systems play a vital role for integrating the PCOC program. Meanwhile, they provided recommendations for enhancing the IT system to better align with their clinical practice. Suggestions included embedding the program directly into the Electronic Medical Records (EMR) system rather than maintaining it as separate entity, visualizing patients’ clinical outcome trends, setting critical scores to trigger alerts, and enabling automatic recommendations for interventions based on captured assessment scores.

##### Incentive systems

The majority of clinicians suggested that establishing appropriate incentives, such as bonuses, souvenirs, or setting positive examples, could motivate them to use the PCOC program.

#### Outer setting domain

##### Local conditions, policies & laws, external pressure

Regarding the external setting factors that either impede or encourage the integration of the PCOC program, opinions among the hospital’s leaders and managers were varied. Some leaders/managers argued that outer settings played no role in the PCOC implementation. As one leader stating that *“I don’t think the integration of the PCOC program in our hospital is connected to the external setting.”*

In contrast, other leaders/managers emphasized that the hospital’s leading role in developing palliative care was a key factor in adopting the PCOC program. One manager pointed out that the pioneering status in palliative care made it the ideal time to implement a standardized program like PCOC, remarking, *“We did not have a standard program for managing quality at the early stage of developing palliative care in our country. It is just the perfect time to implement the PCOC program since our hospital is a leader.”* Clinicians within the unit also echoed that the unit’s leading role in the field of palliative care in Mainland China motivates them to willingly accept the responsibility of embracing new challenges. Additionally, they expressed the need for healthcare insurance to cover the program.

## Discussion

In this qualitative study using the CFIR, we explored the perceived barriers and facilitators among palliative care professionals and hospital leaders regarding the integration of a PCOMs-based quality improvement program – the PCOC model – into routine clinical practice in a palliative care unit in Mainland China. To our knowledge, this is the first investigation into barriers and enablers of an ongoing PCOMs-based quality improvement program in the field of palliative care in mainland China. The findings of this study will provide robust evidence and constructive recommendations for integrating and sustaining a PCOMs-based quality improvement program in Mainland China and other countries where palliative care is still at the early stages of development.

Our findings show that the barriers and facilitators to PCOMs and quality improvement in this hospital in mainland China are similar with Western experiences in palliative care (Antunes et al. 2013; Davis et al. 2023; Lehmann et al. 2021). However, unique to our research are specific barriers and facilitators to the PCOC model, emphasizing the importance of knowledge and self-efficacy in countries where palliative care is in its infancy. New insights include assessment frequency, clinical protocol integration into electronic records, and clinician work numbness – a previously unreported barrier.

In our study, participants identified the PCOC program’s recommended assessment frequency as a major concern, emphasizing the need for dynamic patient assessments to reflect changing palliative care needs while noting potential workload increases. This issue aligns with healthcare professionals’ common concerns about assessment frequency in PCOM-based programs, highlighting the need to balance professional recommendations with patient comfort, especially for palliative care inpatients (Antunes et al. 2013) (Bradshaw et al. 2021). In our earlier study, we tried to assess the test-retest reliability of the PCOC SAS but found that palliative care inpatients felt burdened by twice-daily assessments, admittedly this was for research purposes rather than routine clinical practice (Dai et al. 2024). Within the Australian inpatient setting program assessments occur at a minimum daily (i.e., within a 24-hour period) for direct care models, and at each contact for inpatient consult liaison services, or at the change in care plan or patient/carer needs, and discharge. Our findings showed that there was a need for greater clarity around this and even perhaps the need to adapt this protocol to clinical care within mainland China.

In addition, Clinicians’ perceived lack of knowledge, capacity, and confidence in palliative care hindered the PCOC program’s application in their practice. They expressed uncertainty about appropriate interventions post-assessment with PCOC tools and suggested integrating specific care plans into the Electronic Medical Records System based on assessment scores – a barrier not commonly reported. Currently, palliative care remains underdeveloped in many developing countries, including Mainland China (Connor et al. 2021). With palliative care still emerging in many developing countries, including Mainland China, building a solid foundation in palliative care knowledge and self-efficacy is vital for implementing innovations and ensuring high-quality care (Soikkeli‐Jalonen et al. 2020). Therefore, enhancing training and education for clinicians is crucial, especially where palliative care is underdeveloped.

Previous studies have highlighted the necessity of using patient outcome assessment to inform clinical practice (Bradshaw et al. 2021; Ito et al. 2023), a point that was further emphasized by our study participants. They proposed several key strategies to enhance the utilization of assessment data. These include embedding the PCOC tools into Electronic Medical Records for easy data entry, creating user-friendly dashboards, visualizing trends in assessment results, communicating results during handovers, setting up alerts triggered by assessment scores, generating regular quality report based on the assessment data and providing feedback on care quality by using patient outcomes, et al. These strategies are aimed at fostering a more data-informed and patient-centered approach to managing the palliative care of inpatients and would support the integration of the PCOC information into treatment planning.

Another finding worth noting from this study is clinicians’ numbness toward their work, particularly among senior staff, which emerges as a new barrier. Numbness is an emotional state characterized by indifference or disengagement, often developing as a coping mechanism where clinicians detach emotionally to protect themselves from the ongoing stress or emotional demands of their work (Isbell et al. 2020). This state can arise from various causes, including exposure to high-stress environments and repetitive tasks. Numbness can have a significant impact, leading to a reluctance to embrace new challenges and innovations (Isbell et al. 2020; Kinman et al. 2023). In our study, the numbness toward their work among senior clinicians was a factor that hindered the PCOC program’s integration into routine practice.

## Strengths and limitations

Our study’s strength lies in its two-month pilot of the PCOC program before conducting interviews, offering deep insights into its barriers and facilitators from healthcare professionals’ and leaders’ perspectives. However, participants’ views were confined to an 8-week experience. The CFIR framework helped thoroughly examine the factors affecting PCOC implementation, valuable in contexts where palliative care is nascent. A limitation is the study’s focus on a single hospital, possibly not capturing the full spectrum of challenges in different settings. Future research should broaden its scope to include various environments for a more comprehensive analysis.

## Conclusions

Guided by the CFIR framework, we explored the barriers and facilitators to integrating the PCOC program into routine clinical practice in a cancer hospital in Mainland China. To overcome these barriers, key strategies were pinpointed for effective integration, such as customizing the program to fit the local context, embedding PCOC tools into electronic medical records, offering continuous education and feedback, utilizing PCOC data for assessments, and nurturing a supportive culture. Importantly, enhancing palliative care professionals’ palliative care knowledge, capacity and self-efficacy is a solid foundation for managing quality and delivering high-quality palliative care. This is particularly vital in regions where palliative care is still developing. Additionally, integrating assessment and response protocols into EMR could accelerate palliative care’s development and implementation.

## Recommendations

Based on the findings of our study, we propose the following recommendations:

- Engage with healthcare professionals’ and explore their perspectives before formally implementing a complex intervention into routine clinical practice.
- Enhance healthcare professionals’ palliative care knowledge and self-efficacy in early-stage palliative care countries and regions.
- Refine the PCOC model to align with the local context, including assessment tools, language, and procedures.
- Embed the PCOC model and clinical protocols into existing EMR systems.
- Engage all levels of hospital hierarchy in the implementation process, including leaders, managers, facilitators, and frontline clinicians.
- Provide education about the PCOC model and feed results back to palliative care professionals on a regular basis.
- Implement data-driven quality improvement initiatives to improve care quality continuously.

## Supporting information

10.1017/S1478951525100369.sm001 Dai et al. supplementary material Dai et al. supplementary material

## Supplementary material

The supplementary material for this article can be found at https://doi.org/10.1017/S1478951525100369.
