# Supplementary material for: Health professionals’ and leaders’ views on routine using patient-centered outcome measures in a Chinese palliative care unit: A qualitative study
Source: Palliat Support Care. 2025 Aug 26;23:e151. doi: 10.1017/S1478951525100369 (PMC13166409; doi:10.1017/S1478951525100369)
Supplement: Dai et al. supplementary material [file S1478951525100369sup001.zip › Supplementary Table 2 The Summary Template.docx]

**Supplementary Table 2 The Summary Template**

| **Recording:** □ Audio recording □ Video recording  **Prepared by:**  **Respondent role:** □ Doctor □ Nurse □ Manager/leader  **Interview methods:** □ Focus group interview □ In-depth individual interview |
| --- |
| 1. **Innovation Domain**:   (1) Effectiveness on improving the quality of PC  (2) Relative advantages of the PCOC  (3) Is the PCOC user-friendly?  (4) Changes to the PCOC to align with the current context  **2. Individuals Domain**  (1) Leadership support  (2) Inner facilitator  (3) External facilitator  (4) Knowledge  (5) Attitude  (6) Other individual-related enablers to the PCOC implementation  (7) Other individual-related barriers to the PCOC implementation  **3. Inner Setting Domain**  (1) Internal enablers to the PCOC implementation  (2) Internal barriers to the PCOC implementation  (3) Adapting the existing workflow to accommodate the implementation of PCOC  (4) IT support  (5) Incentive system establishment  **4. Outer Setting Domain**  (1) External enablers to the PCOC implementation  (2) External barriers to the PCOC implementation  **5. Others:** |
