# Supplementary material for: Health professionals’ and leaders’ views on routine using patient-centered outcome measures in a Chinese palliative care unit: A qualitative study
Source: Palliat Support Care. 2025 Aug 26;23:e151. doi: 10.1017/S1478951525100369 (PMC13166409; doi:10.1017/S1478951525100369)
Supplement: Dai et al. supplementary material [file S1478951525100369sup001.zip › Supplementary Figure 1.docx]

**
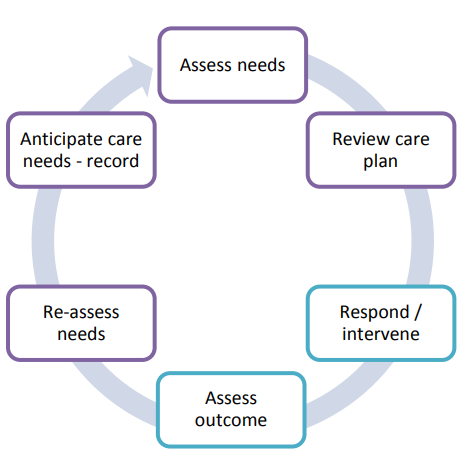
**

**Supplementary Figure 1 PCOC routine clinical assessment and response framework**

Note: The palliative care needs of patients are assessed using five standardized tools:

- PCOC Symptom Assessment Scale (PCOC-SAS) for symptom distress;
- Palliative Care Problem Severity Score (PCPSS) for symptom severity;
- Palliative Care Phase for clinical acuity and urgency;
- The Australia-modified Karnofsky Performance Status (AKPS) for performance status;
- The Resource Utilization Groups - Activities of Daily Living (RUG-ADL) for functional dependency.
