# Supplementary material for: Health professionals’ and leaders’ views on routine using patient-centered outcome measures in a Chinese palliative care unit: A qualitative study
Source: Palliat Support Care. 2025 Aug 26;23:e151. doi: 10.1017/S1478951525100369 (PMC13166409; doi:10.1017/S1478951525100369)
Supplement: Dai et al. supplementary material [file S1478951525100369sup001.zip › Supplementary Table 1 CFIR Informed Interview Guideline.docx]

| **Supplementary Table 1 CFIR Informed Interview Guideline** | | | |
| --- | --- | --- | --- |
| **Interview Guideline** | | | |
| **CFIR Domain** | **Construct(s)** | **Questions** | **Neutral Domain Name** |
| **Innovation Domain** | Innovation Evidence-Base | Please, in your own words – share what you understand the PCOC model to be? What is PCOC and how is it meant to work? Do you think the PCOC model is effective evidence to improve the quality of palliative care? | Effectiveness on improving the quality of Palliative Care |
|  | Innovation Relative advantage | Please, take a moment to think about what you used to do in your service before the PCOC model was introduced. What did you used to do? How does the PCOC model compare to the existing programs on driving the quality of palliative care in your setting?  How does the assessment and response protocol of the PCOC model complement the existing program?  What are the advantages of using the PCOC assessment and response routinely? What are the disadvantages? | Relative advantages  of the PCOC |
|  | Innovation Complexity | How complicated is the PCOC assessment and response protocol? | Is the PCOC User-friendly? |
|  | Innovation Adaptability | What changes do you think need to make to the PCOC assessment and response protocol to strengthen its daily use within the routine clinical practice? | Changes to the PCOC to align with the current context |
| **Outer Setting Domain** | [Local Conditions](https://cfirguide.org/constructs/local-conditions/), [Policies & Laws](https://cfirguide.org/constructs/policies-laws/), [External Pressure](https://cfirguide.org/constructs/external-pressure/) | We regard the local, state, or national performance measures, policies, regulations, or guidelines or peer pressure, the effectiveness of PCOC model on improving the quality of palliative care in other countries/regions et al to be the external factors that may have an impact on the implementation of the PCOC model, what external factors may facilitate the integration of the PCOC assessment and response protocol into the routine clinical practice.  What external factors may impede you from using the PCOC assessment and response routinely? | External barriers and enablers to the PCOC implementation |
| **Inner Setting Domain** | [Structural Characteristics](https://cfirguide.org/constructs/structural-characteristics-updated/), Culture | What internal factors may facilitate the integration of the PCOC assessment and response into the routine clinical practice? We regard the hospital’s or unit’s culture (general beliefs, values, assumptions that people embrace), work infrastructure like IT, your current work processes, PCOC implementation readiness or the characteristics of the patients or their families/carers et al to be the internal factors that may have an impact on the implementation of the PCOC model.  What internal factors may hinder the integration of the PCOC assessment and response into the routine clinical practice? What do you think should be done to overcome these factors?  How can other internal factors be addressed to better embedded the PCOC assessment and response into the routine clinical practice? | Internal barriers and enablers to the PCOC implementation |
|  | Implementation and/or delivery of innovation _ Compatibility | How well do you think the PCOC model will fit with the current working process? Can you describe how the PCOC will be integrated into current processes? | Adapting the existing workflow to accommodate the implementation of PCOC |
|  | Structural characteristics _ Information Technology Information Technology Infrastructure | What kinds of infrastructure changes, such as the IT system, are needed to accommodate the integration of the PCOC assessment and response protocol into the routine clinical practice? | IT support |
|  | Implementation and/or delivery of innovation _ Incentive systems | What incentives/rewards might help ensure the integration of the PCOC assessment and response protocol in the routine clinical practice? | Incentive system establishment |
| **Individuals Domain** | Roles Subdomain _ High-level Leaders, Mid-level Leaders | What level of involvement has leadership at your organization had so far with the PCOC model? How has this level of involvement affected the use of PCOC? | Leadership support |
|  | Roles Subdomain _ Implementation Leads | What’s your view on whether it is necessary to arrange a senior healthcare professional from within your clinical team to be an improvement facilitator? What type of support do you need from an improvement facilitator to help you implement the PCOC model into routine clinical practice? How does the internal facilitators could support the implementation of the PCOC model? | Inner facilitator |
|  | Roles Subdomain _ Implementation facilitators | My current role is an external implementation facilitator for the PCOC model, and my responsibility include introducing the PCOC model, providing the PCOC education session, producing the quality care report and organizing palliative care improvement strategies workshop etc. What other support do you need from an external implementation facilitator to help implement the PCOC model into routine clinical practice? | External facilitator |
|  | Characteristics Subdomain _ Capability | How has your knowledge about the PCOC assessment and response protocol shaped the way you use the PCOC program? What about your beliefs? What are some of the beliefs that you have that may have influenced the way you use the PCOC model? | Knowledge |
|  | Characteristics Subdomain _ Need | What is your attitude towards routine PCOC model use? | Attitude |
|  | Characteristics Subdomain _ Opportunity, Motivation | We’ve discussed a great deal about the PCOC model, but you may have other considerations that you think are influencing the integration of the PCOC model into clinical practice? What other factors have influenced your decision to integrate the PCOC assessment and response into your daily clinical practice?  What weakens your commitment or actions to integrate the PCOC assessment and response into your daily clinical practice? | Other individual-related barriers and enablers to the PCOC implementation |
