# Supplementary figures and images for: Health professionals’ and leaders’ views on routine using patient-centered outcome measures in a Chinese palliative care unit: A qualitative study
Source: Palliat Support Care. 2025 Aug 26;23:e151. doi: 10.1017/S1478951525100369 (PMC13166409; doi:10.1017/S1478951525100369)

**
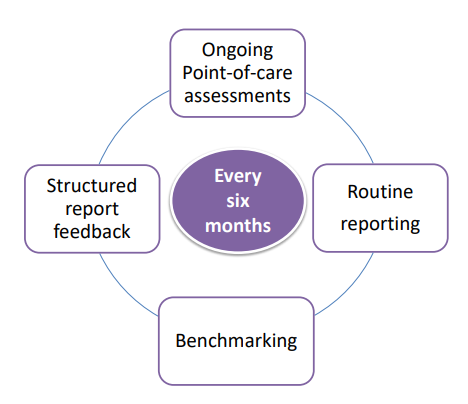
**

**Supplementary Figure 2 PCOC cycle**

Supplement: Dai et al. supplementary material [file S1478951525100369sup001.zip › Supplementary Figure 2.docx]
